# Might synthetic cannabinoids influence neural differentiation?

**DOI:** 10.1080/07853890.2021.1893997

**Published:** 2021-09-28

**Authors:** Evguenia Bekman, Tiago Barata, Cláudia Miranda, Sandra Vaz, Carla Ferreira, Alexandre Quintas

**Affiliations:** a SCERG – Stem Cell Engineering Research Group, IST – Taguspark, Porto Salvo, Portugal; b Instituto de Medicina Molecular, Faculdade de Medicina da Universidade de Lisboa, Avenida Prof. Egas Moniz, Lisboa, Portugal; c Centro de Investigação Interdisciplinar Egas Moniz (CiiEM), Egas Moniz Cooperativa de Ensino Superior, Caparica, Portugal; d The Discoveries Centre for Regenerative and Precision Medicine, Lisbon Campus, IST – Taguspark, Porto Salvo, Portugal; e Laboratório de Ciências Forenses e Psicológicas Egas Moniz, Campus Universitário – Quinta da Granja, Caparica, Portugal

## Abstract

Phytocannabinoids are psychotropic substances found in cannabis that bind to the endocannabinoid receptors regulating a variety of physiological processes in human body, including synaptic activity in the central nervous system and metabolic effects in the peripheric nervous system among many others [1,2]. Synthetic cannabinoids emerged as popular alternative to cannabis. Most of these substances are synthetic analogues of Δ^9^-THC, the psychotropic compound of cannabis, binding with higher affinity to the endocannabinoid receptor CB_1_ and eliciting a stronger and long-lasting effect on brain cells. Molecular structure of synthetic cannabinoids is always changing escaping the control by authorities and increasing the hazard for general population. The popularity of cannabis and its derivatives may lead, and often does, to child’s exposure to cannabinoids both in utero and through breastfeeding by a drug-consuming mother. Prenatal exposure to cannabis has been associated with higher risk of newborn morbidity [2,3], altered rate of mental development and significant changes in nervous system functioning [4,5]. However, direct evidence that these effects are mediated through the binding of cannabinoids to endocannabinoid receptors is still lacking. Thus, it is paramount to better understand the psychoactive effects of natural and synthetic cannabinoids on the developing human brain.

We conveyed a pilot study in which human induced pluripotent stem cells (hiPSCs) were induced into neural differentiation and treated with a non-psychotropic component of cannabis, cannabidiol, known to bind the CB_2_ receptor, and two synthetic Δ^9^-THC analogues, THJ-018 and EG-018. Neuronal differentiation and functional maturation were assessed by immunofluorescence, qRT-PCR and single cell calcium imaging. Our results indicate that all three substances have profound impact on the differentiation, maturation and functioning of developing CNS neurons, providing a new evidence for the importance of thorough research of the impact of pre-natal exposure to natural and synthetic cannabinoids.
